# Machine learning-enhanced monitoring of global copper mining areas

**DOI:** 10.1038/s41597-025-05296-y

**Published:** 2025-07-01

**Authors:** Houxuan Li, Peng Wang, Tim T. Werner, Bin Chen, Wei-Qiang Chen

**Affiliations:** 1 Institute of Urban Environment, Chinese Academy of Sciences, 1799 Jimei Road, Xiamen, 361021 China; 2 School of Geography, University of Melbourne, 221 Bouverie Street, Carlton, VIC 3010 Australia; 3 Department of Environment Science and Technology, Fudan University, 220 Handan Rd., Shanghai, 200433 China

**Keywords:** Environmental monitoring, Environmental impact

## Abstract

Copper is one of the most critical minerals for the global transition to low-carbon energy. However, as copper mining activities expand worldwide, they often result in significant environmental impacts, yet the monitoring approaches and up-to-date databases remain limited. In this study, we present a high-resolution, site-specific database of global copper mining activities, developed using a machine learning approach that leverages Earth observation images and various dispersed data sources. Our database encompasses approximately 1,313 copper mines, covering an area of 7,267 km^2^, and includes detailed monitoring of operational land use categories such as open pits, waste rock dumps, and tailings storage facilities as of 2022. Additionally, we analyse land use intensity at each mine site based on inferences of copper production levels to facilitate comprehensive comparisons and improved management strategies. This database can help to reveal the adverse impacts of copper mining behind the energy transition. The dataset is available for download from 10.6084/m9.figshare.28680863.v1.

## Background & Summary

Copper, as one of the energy transition minerals (ETMs)^1–3^, is critical to nearly all low-carbon technologies ranging from electric vehicles (EVs)^4^, charging infrastructure, copper electric wire, solar photovoltaics (PV)^5^, wind^6^ and batteries. As one of most widely used metals, copper demand is expected to at least double by 2050 to support intensified electrification pathways^7^. This may boost future global copper mining and their associated environmental and social impacts to unprecedented scales. Hence, given that all mine sites are not alike, there is a need to establish a site-specific copper mine database to support research on the environmental impacts caused by future demand.

Among all environmental indicators, the land use of mining is one of the key parameters used to describe the scale of copper mining production^8^, ecological environmental impact, and pressure on ecological diversity^9–12^. These indicators are widely applied in the assessment of copper mining impacts on biodiversity^13,14^, its influence on the supply chain and sustainable resource utilization^15–17^, mine disaster risk management^18,19^. However, enormous data gaps prevent the progress of these studies, primarily due to limited transparency in the mining industry^20^.

Remote sensing monitoring of copper mining areas presents distinct technical complexities beyond conventional land cover mapping, primarily due to three interrelated challenges: the spectral confusion between mining features and surrounding environments, the dynamic spatial-temporal evolution of mining footprints, and the geographic heterogeneity of spectral signatures. Copper mining operations integrate diverse land-use components—open-pit excavations, waste rock accumulations, tailings storage facilities, processing plants, and transport networks—which exhibit near-identical spectral responses to adjacent bare soils, quarry sites, and urban constructions, particularly in arid/semi-arid regions where vegetation masking effects are minimal^21^. Simultaneously, rapid spatial expansions often outpace the temporal resolution of moderate-frequency satellite systems like Landsat, leading to temporal baseline mismatches in traditional monitoring approaches. Compounding these issues, latitudinal variations in weathering processes and climatic conditions induce substantial intra-class spectral heterogeneity, rendering globally uniform classification thresholds ineffective—a limitation exacerbated by the lack of geographically adaptive algorithms in existing methodologies^22^.

Previous remote sensing investigations of mining areas have predominantly depended on human-driven vectorization processes for satellite image interpretation^23–26^, a methodology fundamentally constrained by three interlinked shortcomings: interpreter-induced variability in boundary delineation, inadequate differentiation of land-use taxonomies, and chronologically inconsistent basemap compilation. The accuracy of extracted mining footprints exhibits operator-dependent discrepancies due to subjective feature recognition thresholds, particularly evident in complex terrains where open-pit mines, waste rock dumps, and tailings ponds exhibit spectral ambiguities with surrounding anthropogenic landscapes. Compounded by the absence of standardized classification protocols, manual approaches often generate generalized polygonal extents that fail to resolve critical intra-site land-use distinctions, thereby limiting their utility in spatially explicit environmental impact assessments^27^. Furthermore, the prevalent practice of temporal mosaicking—aggregating multi-year image composites from heterogeneous sources (e.g., merging 2010–2020 Landsat archives), a dynamic process optimally monitored through high-frequency, sensor-consistent observation systems.

Currently, several global mining land-use datasets are available, such as those developed by Maus *et al*.^23^ and Tang *et al*.^24,25^. Maus *et al*.‘s dataset mainly relies on manual interpretation methods, broadly covering various mining commodities but lacking precise delineation and commodity-specific details, particularly for copper mining operations. Tang *et al*. provided detailed spatial delineations; however, their dataset faces temporal consistency issues due to the heterogeneous nature of satellite sources and image acquisition times. Moreover, although their studies covered numerous mining sites, these delineations were not directly integrated with operational or commodity-specific data, limiting their applicability for detailed analyses of environmental impacts specific to copper mining. Consequently, existing datasets fail to adequately combine temporal consistency, accurate automated delineation, and commodity-specific operational information necessary for detailed environmental assessments and management decisions.

With the emergence of increasing high-resolution Earth observation data and new machine learning methods, the extraction of land use in copper mining areas through remote sensing techniques has become feasible^26–30^. Through machine learning, copper mining areas can be distinguished from other surrounding features, thereby extracting the land use of copper mines. However, few studies have produced a comprehensive database of the land use area of copper mines on a global scale.

In this study, we extracted copper mine land use area around the world. We determined the location and scope of copper mines based on databases provided by Standard & Poor’s(S&P)^31^, USGS, and previous studies^32^, and acquired multispectral remote sensing images from the Sentinel-2 satellite for the year 2022. We distinguished copper mining areas from other surrounding features based on their unique remote sensing image characteristics and established a random forest model to automatically classify the land use area of each mine. This distinguishes our study from previous works as the first machine learning classification of mine footprints performed for a commodity at a global scale.

Our database covers 1,313 copper mines worldwide, and spans an area of 7,267 km^2^. Our database provides a detailed description of the global copper mine land use area in 2022, and the overall accuracy of the remote sensing model reached 91.08% (for details see the section on Technical Validation). We also obtained data on copper mines, including commodity production, production capacity, start and end dates of mining, and calculated the historical total production of each copper mine. We inferred the unit area mining intensity of the copper mines, providing a detailed statistical foundation for future risk assessments of copper mining areas.

## Methods

### Data processing and modeling workflow

**Fig. 1:**
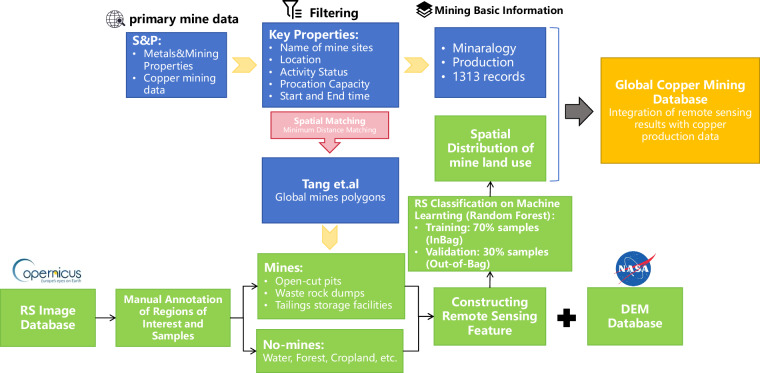
Data Processing Workflow.

Figure 1 illustrates the entire methodological pipeline used in this study, comprising data integration, satellite imagery preprocessing, classification model development, and data validation. Initially, mine locations and attributes from the S&P databases were integrated and matched with mine footprint polygons provided by Tang *et al*.^25^. Sentinel-2 satellite imagery from the year 2022 was preprocessed on the Google Earth Engine platform to obtain cloud-free image composites. Subsequently, spectral indices (NDVI, NDWI, BSI, EVI and IBI) and Digital Elevation Model (DEM) data were calculated and incorporated as inputs for training a Random Forest classification model. Finally, the model’s performance was evaluated using a confusion matrix calculated from validation samples, and the overall accuracy, user’s accuracy, producer’s accuracy, and Kappa coefficient were derived.

### Mine site data sources and management

To identify land use areas of global copper mines in satellite imagery, we must first ascertain the locations of these mines. Current global mining databases primarily consist of point data, which includes information from S&P^31^ and the United States Geological Survey (USGS), among others. The characteristics of such data are such that they only record the coordinates of mining areas without delineating the spatial extent of these areas. These point-source data include some additional records on operational characteristics like the mining area category, ownership information, production information, and capacity information.

While previous studies have sought to map the spatial extent of mining areas, creating databases of land use within these areas, they have not recorded operational information about the mining areas, merely depicting the range of land use in mining regions. As such, there is a need to effectively connect the ‘point source’ operational data, with available ‘footprint’ data sources. We procured copper mine point data disseminated by S&P and USGS, as well as global mining area land data published by Tang *et al*.^25^. These two distinct types of data were spatially connected and matched. The original data of point data is in tabular form. We converted it into vector format spatial point features, and then transformed these point features and all coordinate data of Tang & Werner’s surface features into the GCS_WGS_1984 projection. Next, using the principle of minimum distance, we spatially link the point features and surface features, matching the rich mining area information from the point feature data to the surface features. Considering that the mining area boundaries delineated by the global mining area land data are not entirely precise, we established a 1 km buffer zone based on the original mining area boundaries to ascertain the recognition scope for classifying land use area within copper mines. Specifically, the spatial matching process was conducted in ArcGIS by applying the spatial join tool using the ‘nearest distance’ option, thus ensuring that each mine site point was accurately associated with its corresponding polygonal mine footprint. The created 1 km buffer effectively accounted for potential positional inaccuracies in polygon boundaries, providing a robust spatial extent for accurate land-use classification. This integration allowed us to precisely combine operational details (production capacity, status, and timelines) from the point-source databases with spatially explicit mining footprint boundaries, significantly improving the reliability of subsequent analyses.

### Satellite data sources and pre-processing

We used Sentinel-2 satellite imagery to classify and analyse copper mine areas. Sentinel-2 is a high-resolution spectral imaging satellite^32^, first launched by the European Space Agency (ESA) on June 23, 2015. It is a high-resolution terrestrial observation mission of the ESA, possessing excellent spatial and spectral resolution capabilities. The Sentinel-2 satellite system includes Sentinel-2A and Sentinel-2B, two satellites located on the same operational orbit with a phase difference of 180°. The revisit period is 5 days, and for mid-high latitude areas, the revisit period is only 3 days, enabling comprehensive and varied terrestrial observation records. The images provided by Sentinel-2A and Sentinel-2B are identical. The satellite sensor encompasses 13 spectral bands, with a spectral range of 443–2203 nm, providing rich spectral information for identifying and land use area within copper mines and different land objects in their surrounding environment. The spatial resolution of the satellite imagery, after preprocessing, can reach 10 m. This implies that, in addition to large-scale copper mines, land use area of medium and small-scale copper mines can also be accurately extracted.

In order to eliminate the interference of clouds in remote sensing images, we collected a series of images from summer to autumn for the mining area, and synthesized a new image by using the median value of pixel values in the image series. Through this method, we obtained cloud-free global remote sensing imagery from Sentinel-2 for the year 2022, with the aim of conducting remote extraction of the global land use area of copper mines. This process was carried out via the Google Earth Engine (GEE) platform^33^.

### Remote sensing classification of land-use types of copper mining areas

Copper mine areas consist of land-use types such as open-cut pits, waste rock dumps, and tailings storage facilities^8^. The extent of each land-use types can be extracted through automated remote sensing classification methods. Among these land-use types:

Open-cut pits are the primary source of ore, and the size of the pit typically reflects the scale of copper mining production.

Waste rock dumps represent the disposal areas of solid overburden removed from a pit or underground shaft in order to access and orebody. In terms of spatial footprint, they are often the fastest changing component within the mine site.

Tailings storage facilities are where the wastes generated after copper ore beneficiation are stored. They contain a mixture of liquid, sludge and solid materials that typically occupy a larger area to promote evaporation, as liquid wastes pose a risk of breach and contamination in the event of dam failure. Due to their large quantity and the presence of temporarily un-processable or harmful components, tailings pose a significant ecological risk^34^.

**Fig. 2:**
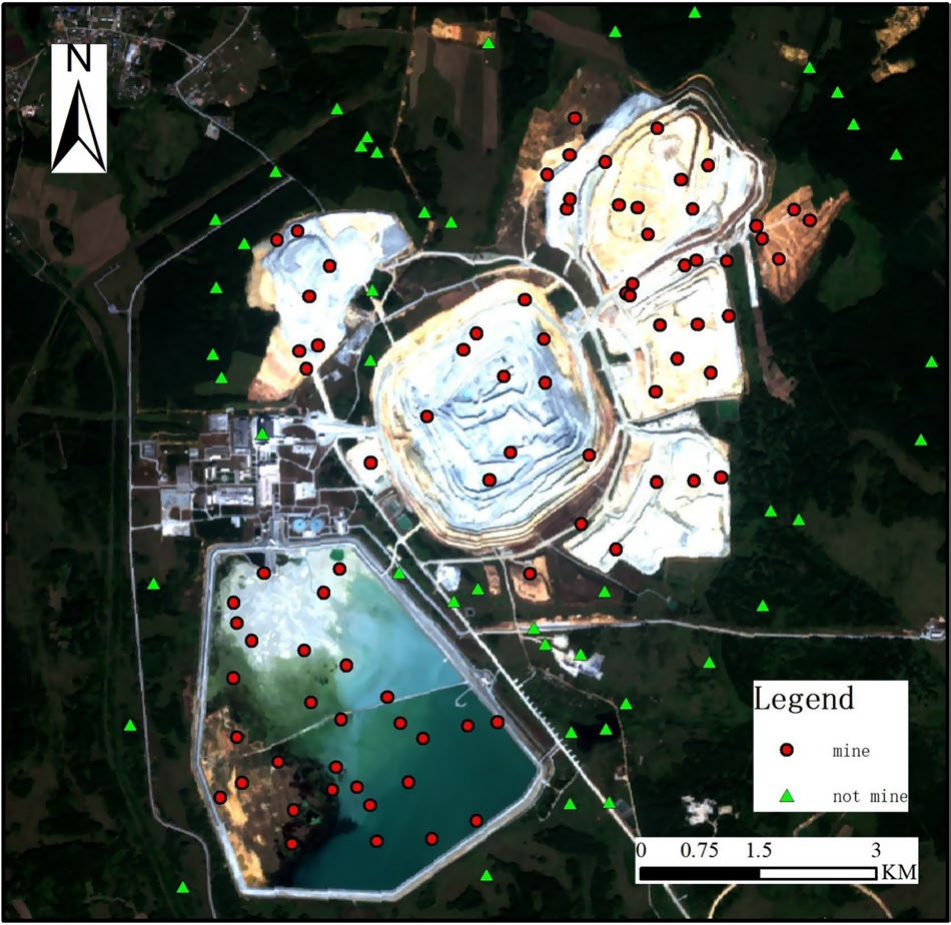
Selection of Copper Ore Sample Points: A Case Study of Dexing Copper Mine in China(Sentinel-2 image date 27 August 2022; approx. location 54° 57′ 8.16″ N, 61° 43′ 4.37″ E).

These land-use types constitute the largest parts of a copper mining area, but other features are also common, including mineral processing infrastructure zones and connecting roads. Their size also indicates the scale of copper production, and each has unique remote sensing image characteristics. As shown in Fig. 2, we have selected sample points for copper mines and non-copper mines on the remote sensing images to train image processing algorithms for machine learning. Based on the previous datasets of the mining area, we conducted sample point collection. Sample points were randomly selected both within the boundary of the mining area and in the surrounding external regions. This process was completed using ArcGIS. To ensure representativeness and accuracy of the training dataset, we employed a stratified random sampling strategy within ArcGIS. Positive sample points (copper mining land-use) were randomly selected across multiple mining land-use classes such as open-cut pits, waste rock dumps, and tailings storage facilities, covering various geographical regions globally. Negative sample points (non-mining features) were randomly distributed in surrounding landscapes including water bodies, forests, croplands, and other non-mining areas adjacent to the mine sites.

Although several global land cover products (e.g., ESA CCI-LC with 300 m resolution, MODIS MCD12Q1 with 500 m resolution) exist and have been widely utilized in global land-cover monitoring, their coarse spatial resolutions do not adequately capture the fine-scale features specific to mining operations^35^. Furthermore, these products typically do not include explicit classification categories for mine-specific land-use types such as open-cut pits, waste rock dumps, and tailings storage facilities. Consequently, they cannot provide the precision required for assessing detailed mine-scale environmental impacts. To overcome these limitations, our study utilizes high-resolution Sentinel-2 satellite imagery (10 m resolution), integrating specific remote sensing indices (NDVI, NDWI, BSI, EVI and IBI) and Digital Elevation Model (DEM) data to ensure the accurate identification and extraction of mine-specific land-use categories. This methodological approach provides significant advantages in capturing detailed spatial characteristics and filling the gaps left by conventional global land cover products

### Classification of copper mining land-use types

Copper mines are distributed globally across various terrains, including forests, mountains, and deserts. Open-cut pits, waste rock dumps, and tailings storage facilities exhibit unique spectral characteristics in optical remote sensing imagery, significantly differing from surrounding features such as vegetation, bare soil, and buildings. To accurately identify and extract these land-use types of copper mines, we used band values as model inputs and also calculated the Normalized Difference Vegetation Index (NDVI)^36^, the Normalized Difference Water Index (NDWI)^37,38^, the Bare Soil Index (BSI)^39^, the Enhanced Vegetation Index (EVI)^40^. and the Index-Based Built-up Index (IBI)^41^ for each land-use type. This approach was designed to mitigate the impact of surrounding forests, rivers, and bare soil on the remote sensing classification of land-use types within the copper mining areas. The formulas for these remote sensing indices are as follows:

1 $${\rm{NDVI}}=\frac{{\rm{NIR}}-{\rm{Red}}}{{\rm{NIR}}+{\rm{Red}}}$$

2 $${\rm{NDWI}}=\frac{{\rm{Green}}-{\rm{NIR}}}{{\rm{Green}}+{\rm{NIR}}}$$

3 $${\rm{BSI}}=\frac{({\rm{SW}}{\rm{IR}}1+{\rm{Red}})-({\rm{NIR}}+{\rm{Blue}})}{({\rm{SW}}{\rm{IR}}1+{\rm{Red}})+({\rm{NIR}}+{\rm{Blue}})}$$

4 $${\rm{EVI}}=2.5* \frac{{NIR}-{Red}}{6* {Red}-7.5* {Blue}+{NIR}+1}$$

5 $${\rm{IBI}}=\frac{2\ast \frac{{\rm{SWIR}}1}{{\rm{SWIR}}1+{\rm{NIR}}}-\left(\frac{{\rm{NIR}}}{{\rm{NIR}}+{\rm{RED}}}+\frac{{\rm{GREEN}}}{{\rm{GREEN}}+{\rm{SWIR}}1}\right)}{2\ast \frac{{\rm{SWIR}}1}{{\rm{SWIR}}1+{\rm{NIR}}}+\left(\frac{{\rm{NIR}}}{{\rm{NIR}}+{\rm{RED}}}+\frac{{\rm{GREEN}}}{{\rm{GREEN}}+{\rm{SWIR}}1}\right)}$$

In the equation, NIR refers to the Near Infrared band, using the reflectance of Sentinel-2’s B8, Red refers to the Red band, using the reflectance of Sentinel-2’s B4, Green refers to the Green band, using the reflectance of Sentinel-2’s B3, Blue refers to the Blue band, using the reflectance of Sentinel-2’s B2, and SWIR1 refers to the Shortwave Infrared band, using the reflectance of Sentinel-2’s B11.

Additionally, this study incorporated a Digital Elevation Model (DEM) as a reference for topographic information of copper mines, aiming to improve the accuracy of the remote sensing classification for land use area within global copper mines.

### Land use classification model development

The Random Forest (RF) algorithm was employed to classify land-use types within copper mine boundaries^42–45^. This choice leverages RF’s advantages over single-model approaches, including its inherent resistance to overfitting and improved generalization performance via ensemble voting of multiple decorrelated decision trees^46–48^. Specifically, the RF model was trained using spectral bands from Sentinel-2 images (including visible, near-infrared, and shortwave infrared bands), remote sensing indices (NDVI, NDWI, BSI, EVI and IBI), and elevation data (DEM) as input variables. To construct the RF model, we randomly divided our manually annotated sample points into training (70%) and validation (30%) datasets using the random partition function in Google Earth Engine. The model was composed of multiple decision trees, with each tree built from bootstrapped subsets of the training data. At each decision node, the optimal splitting feature was determined using the Gini impurity criterion. To ensure robustness and accuracy, the model’s performance was evaluated using an independent validation dataset, and accuracy metrics—including the overall accuracy, user’s accuracy, producer’s accuracy, and Kappa coefficient—were calculated through a confusion matrix.

### Evaluating copper mining land-use intensity

To calculate the exploitation intensity of each copper mine we also incorporated mine site operational statistics. Due to the diverse geographical environments, ore grades, start and end times of mining, as well as geopolitical factors in different regions where each copper mine is located, we defined a parameter to characterize mining intensity, termed the unit area mining intensity index. By determining the production capacity of each copper mine and the start and end years of mining, we estimated the historical total production, and then based on the land use area of each mining area, calculated the copper mining intensity per unit area. The copper mine production capacity data is sourced from the Standard & Poor’s Capital IQ Pro database.

### Data collection and attribute assignment

The vectorized copper mining areas were derived from Sentinel-2 classification results and manually refined to ensure boundary accuracy. Each polygon in the vector data contains the following attributes:

- **Property_Name:** Unique identifier of the mining site (e.g., “Escondida Copper Mine”).
- **Latitude/Longitude:** Centroid coordinates of the mining area (WGS84 geographic coordinate system).
- **Primary_Commodity:** Dominant mineral extracted (e.g., “Copper”).
- **List_of_Commodity:** Secondary minerals associated with the site (e.g., “Gold, Molybdenum”).
- **Activity_Status:** Operational status categories (e.g., “Active”, “Inactive”, “Under Development”).
- **Country/State_Province:** Administrative region of the mine.
- **Land_use:** Land cover type classified by the RF model.
- **Cumulative_Production:** Total historical production (metric tons) calculated from annual capacity and operational years.
- **Mining_Intensity:** Production density (ton/km^2^) as Cumulative_Production / Polygon_Area

Attribute values were cross-validated against S&P Global and USGS records to ensure consistency with reported mining activities.

## Data Repository Structure

The dataset “Machine learning-enhanced monitoring of global copper mining areas” and associated code are hosted in the figshare repository (10.6084/m9.figshare.28680863) with the following organization:

- Vector data:
  Global_Copper_Mines_2022.shp: Shape file containing all mining area polygons with attributes.
- GEE Code:

code.txt: Google Earth Engine (GEE) script for RF model training and classification.

## Data Records

The full dataset, titled “Machine learning-enhanced monitoring of global copper mining areas”, is publicly available on the figshare repository^49^. The dataset contains vectorized land-use boundaries for 1,313 copper mines globally, with a total mapped area of 7,267.1 square kilometers. All land-use areas are expressed in square meters (m^2^) in the attribute table. The repository includes shapefiles of mining boundaries and the code used for classification, allowing users to fully replicate and analyze the dataset.

### Remote sensing data of copper mining area

In previously published copper mine land databases by other experts or institutions, the delineated areas included not only the various land-use types of copper mines, but also nearby forests, roads, bare land, etc. In other words, these databases only sketched the approximate range of the copper mines without precisely extracting the mining areas. Figure 2 shows the copper mine land use area obtained through our remote sensing method. The left part of the image is the Sentinel-2 remote sensing image of the identified area, while the right part is the land use of the copper mine area. We have accurately identified the range of the copper mine itself, strictly distinguishing and eliminating nearby features from the mining area. Additionally, we have accurately extracted the land use area of the copper mine area in different geographical environments, including deserts, Gobi, forests, etc.

**Fig. 3:**
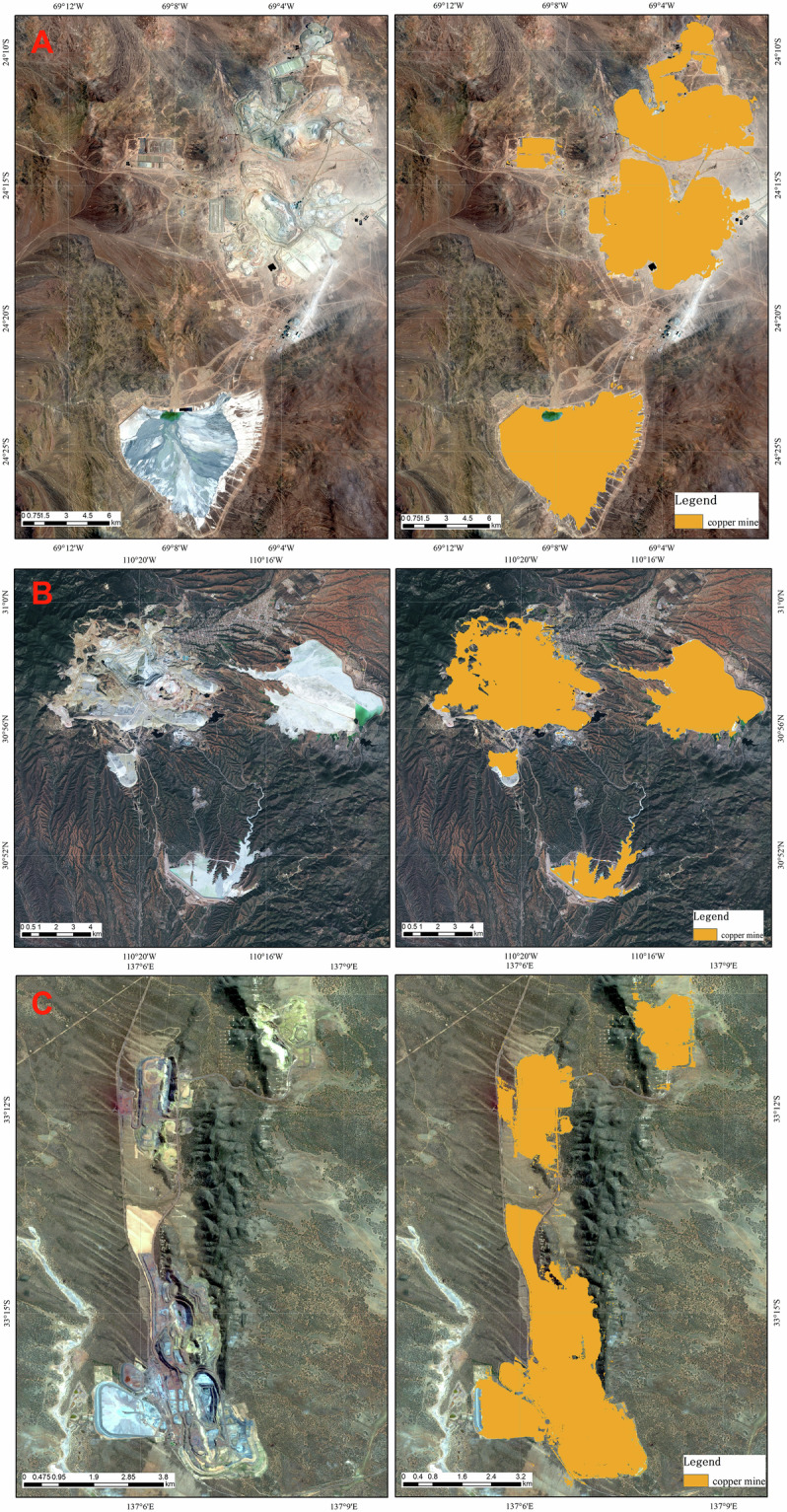
Example of remote sensing extraction of land use area from copper mine. (**A**) Escondida and Zaldivar copper mines, approx. location 24° 16′ 39.27″ N, 69° 3′ 23.14″ W; (**B**) Buena Vista copper mine, approx. location 30° 56′ 51.40″ N, 110° 18′ 16.97″ W; (**C**) Olympic copper mine, approx. location 33° 15′ 21.51″ S, 137° 6′ 33.08″ E.

Figure 3(A) shows the Escondida and Zaldivar copper mines located in the Atacama Desert in northern Chile, and Escondida is the largest copper mine in the world in terms of annual production, surrounded by rocks and desert with little vegetation cover. As the features within the copper mine area are mostly bare land and rocks, this poses a challenge for mine area extraction. Figure 3(B) shows the Buena Vista copper mine located in Mexico, which is distributed in the mountains and surrounded by many residential areas. The rivers within the mountainous area can also interfere with the model’s classification. Yet, our model not only distinguishes the feature differences between surrounding vegetation, residential areas, water surfaces, and mining areas, but also accurately differentiates the rocks and desert in the surrounding area from the open pits, waste rock piles, and tailings reservoirs in the mining area, and extracts the range of the mining area. For smaller-scale copper mines, as shown in Fig. 3(C) for the Olympic copper mine in Australia, the mining area of the copper mine is still accurately extracted.

### Overview of global copper mines and land use area

**Fig. 4:**
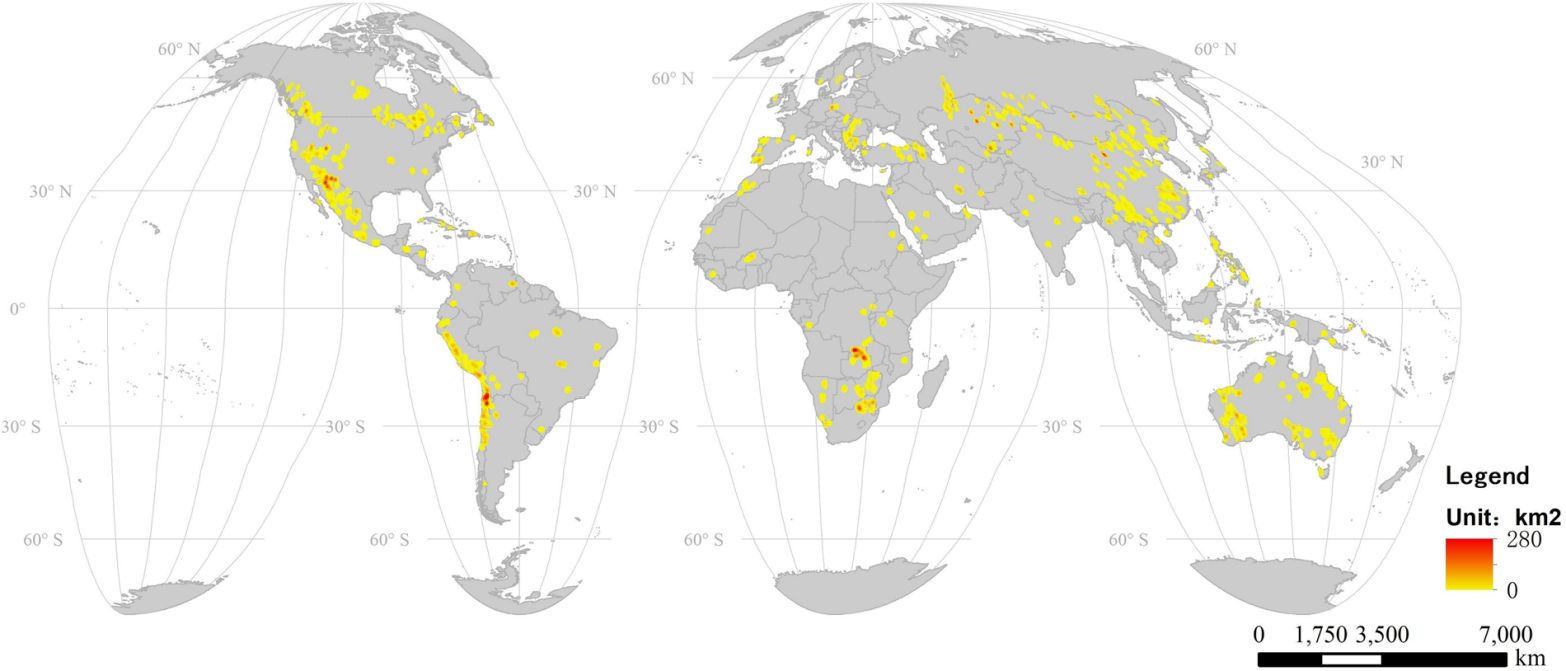
A Kernel Density Analysis of Global Copper Mining Land Use Area.

**Fig. 5:**
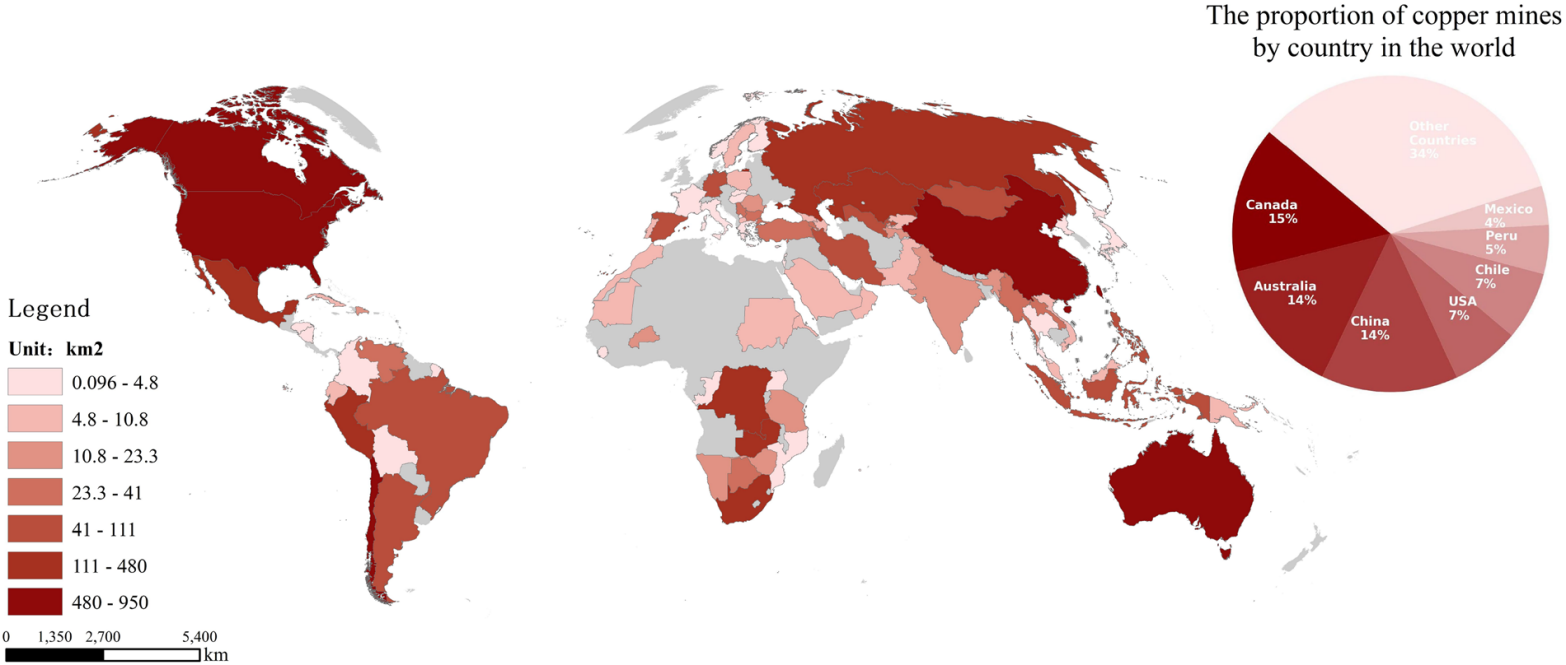
Spatial statistics of the land use area of copper mines in countries around the world.

This study monitored a total of 1,313 copper mines and spans an area of 7267.1 km^2^ worldwide across 80 countries. Figure 4 illustrates the geographical distribution of the copper mining land use area obtained on a global scale. A kernel density analysis was conducted on the global copper mining land use area derived from remote sensing, defining the distribution of the within a 25 km x 25 km grid.We can see that Canada, Australia, China, the United States, Chile, Peru, and Mexico account for 66% of the world’s copper mines. Among them, Canada, Australia, and China have far more copper mines than other countries, with these three countries alone accounting for 43% of the world’s total. The Fig. 5 also displays the land use area of copper mines in various countries around the globe. The results show that although the number of copper mines in Chile is much lower than in Canada, Australia, and China, it has the largest area of copper mine land use. This indicates that the average scale of Chile’s copper mining is enormous. Despite the number of copper mines being only half or even less than that of Canada, Australia, and China, it still has the third-largest copper mine land use in the world. Australia accounts for 14% of the world’s copper mines, ranking second globally, but its copper mine land use area is significantly smaller than that of China, Chile, Canada, and the United States. This suggests that the average land use area of Australia’s copper mines is smaller, and the number of large-scale copper mines is fewer. A similar situation is also found in Canada. While Canada ranks first globally in the number of copper mines, its copper mine land use area only ranks fourth worldwide, nearly 200 km^2^ less than that of Chile, which ranks third.

### Overview of copper unit area mining intensity

**Fig. 6:**
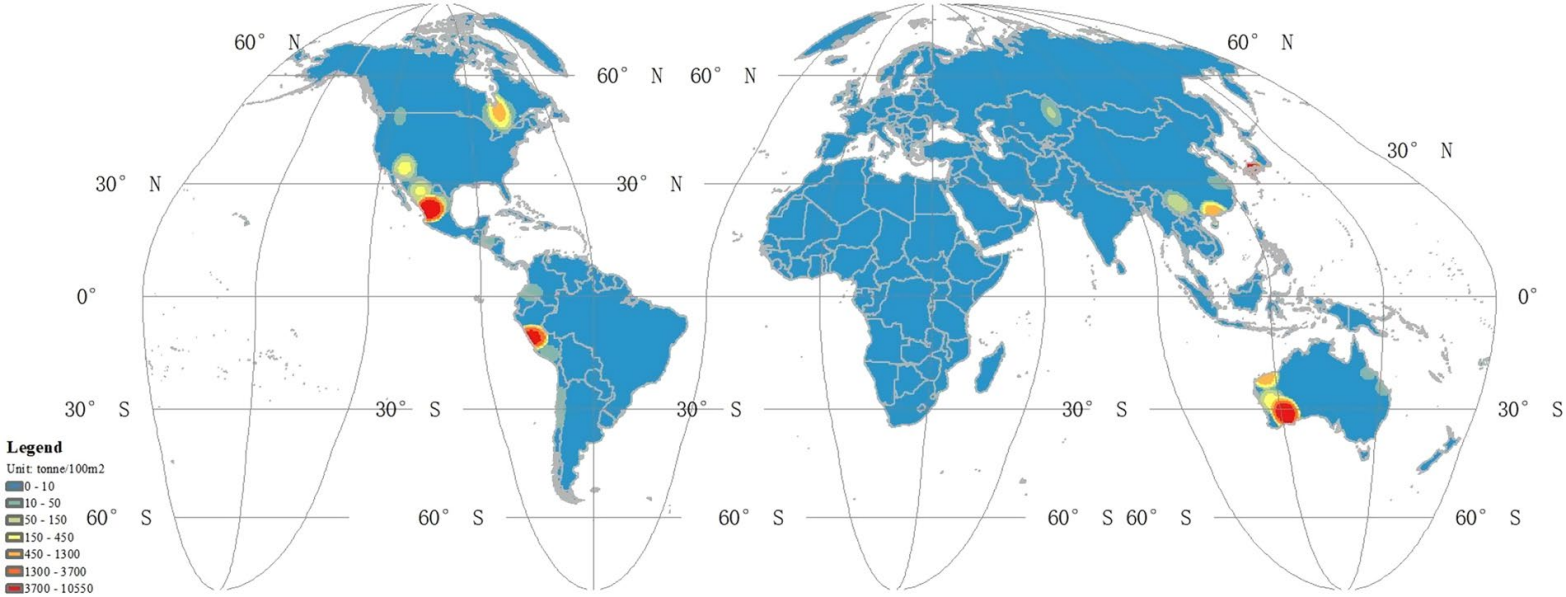
The unit area mining intensity of global copper mine.

We spatially linked the unit area mining intensity index of each copper mines with the polygon of each copper mine in the database. The mining intensity of different copper mines can be referenced in the attribute table of the vector file. To facilitate better display, we conducted a kernel density analysis on copper mining intensity. Figure 6 illustrates the spatial variations in copper mining intensity. The results indicate that areas with high unit mining intensity are mainly distributed in the southwest of Australia, northern Mexico, and Peru. In other words, copper mines in these regions produce more copper per unit area of land, implying that these areas pose a greater ecological threat due to copper mining activities.

### Spatial and attribute validation

The vector dataset contains 1313 globally distributed copper mining areas (as of 2022), each rigorously annotated with 11 attributes (Table 1). Key data generation steps include:

- Spatial Boundaries: Mining polygons were vectorized from the RF classification results, with centroids calculated for coordinate assignment.
- Attribute Sources: Primary_Commodity, Activity_Status, Cumulative_Production and List_of_Commodity were cross-validated against the S&P Global Market Intelligence Database and annual reports from the USGS Mineral Commodity Summariesto ensure mineralogical accuracy.
- Land_Use were manually confirmed via Land use Remote Sensing classification model.
- Spatial Consistency: All coordinates follow the WGS84 geographic coordinate system (EPSG:4326), and mining intensity (Mining_Intensity) was standardized as cumulative production divided by polygon area (automatically calculated via Arcgis field calculator).

**Table 1:** Summary of Vector Dataset Attributes (Take Escondida in Chile for example).

Field Name	Data Type	Description	Example Value
Property Name	Text	Official name of the mining site	Escondida
Latitude	Double	Latitude of the mine centroid (WGS84, decimal degrees)	−24.26889
Longitude	Double	Longitude of the mine centroid (WGS84, decimal degrees)	−69.07466
Primaray Commodity	Text	Dominant mineral extracted	Copper
List of Commodity	Text	Associated minerals (comma-separated)	Copper, Gold, Silver
Activity Status	Text	Operational status classification: e.g., Active/Inactive	Active
Country	Text	Country where the mine is located	Chile
State/Province	Text	State or province of the mine location	Antofagasta
Land Use	Double	Land area altered by mining activities (unit: m^2^)	46538704.12
Cumulative Production	Double	Total historical production (metric tons)	37298099.17
Mining Intensity	Double	Production density (Cumulative_Production / polygon area, units: 100 m^2^/tons)	80.14425815

## Technical Validation

### Model accuracy and validation

In this study, the accuracy of the classification was verified using a confusion matrix, and the overall accuracy, user’s accuracy, producer’s accuracy, and Kappa coefficient were calculated to represent the results in the confusion matrix^50^. The overall accuracy refers to the percentage of correctly classified samples out of all samples, i.e., the sum of all values on the diagonal of the confusion matrix divided by the total sum of all samples. The user’s accuracy refers to the ratio of samples that fall into a certain category on the classification map and are correctly classified into that category. The producer’s accuracy refers to the probability of correctly classifying the ground truth reference data for that category. The Kappa coefficient is an indicator to measure classification accuracy. Generally, the Kappa coefficient ranges from 0 to 1, and a higher Kappa coefficient indicates higher classification accuracy. The specific calculation formulas are as follows:

6 $${Overall\; Accruacy}=\frac{\mathop{\sum }\limits_{i=1}^{n}{P}_{{ii}}}{N}\times 100 \% $$

7 $${User}\mbox{'}s\,{Accuracy}=\frac{{P}_{{ii}}}{{P}_{i+}}\times 100 \% $$

8 $${Producer}\mbox{'}s\,{Accuracy}=\frac{{P}_{{ii}}}{{P}_{+i}}\times 100 \% $$

9 $${Kappa}=\frac{N\mathop{\sum }\limits_{i=1}^{n}{P}_{{ii}}-\mathop{\sum }\limits_{i=1}^{n}({P}_{i+}\times {P}_{+i})}{{N}^{2}+\mathop{\sum }\limits_{i=1}^{n}({P}_{i+}\times {P}_{+i})}$$

where *n* represents the number of columns in the confusion matrix; *P*_*ii*_ denotes the number of pixels in the *i*th row and *i*th column of the confusion matrix, representing the number of correctly classified instances; *P*_*i+*_ and *P*_*+i*_ respectively represent the total number of pixels in the *i*th row and *i*th column; *N* stands for the total number of pixels used for validation.

### Validation data selection

**Fig. 7:**
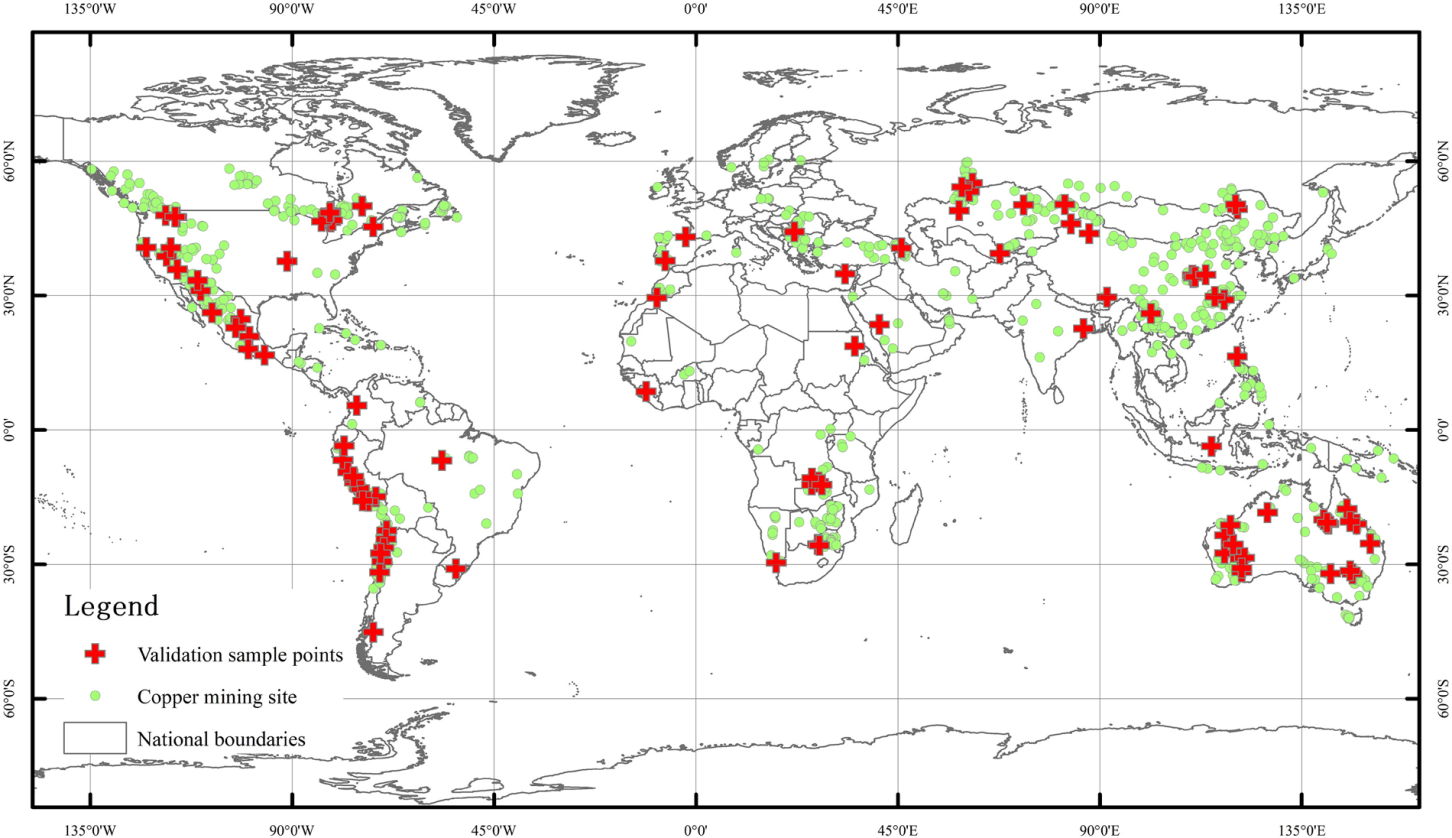
Spatial distribution of validation sample points used for accuracy assessment.

To rigorously evaluate the classification accuracy of our remote sensing model, we performed an independent validation based on manual interpretation of mining boundaries at 100 randomly selected copper mine sites. These sites were chosen to ensure broad geographic, ecological, and operational diversity. Specifically, the selected mines are distributed across six continents and span a variety of environmental settings, including arid deserts, tropical forests, temperate mountain regions and savannahs. The selected sites also represent a wide range of mine scales, ore grades, and operational statuses, thereby capturing the heterogeneity of global copper mining landscapes.he spatial distribution of these validation sample points is illustrated in Fig. 7.

For each of the 100 mine sites, we conducted manual delineation of the actual mining boundaries through visual interpretation of 2022 Sentinel-2 imagery using ArcGIS. The interpretation process relied on a combination of spectral reflectance characteristics, textural patterns, shape morphology, and contextual cues. Interpreters paid particular attention to distinguishing mining-specific features—such as open-pit outlines, waste rock dumps, and tailings storage facilities—from adjacent bare land or infrastructure. Pixels located strictly within the manually delineated polygons were labeled as “Copper mine” class, while pixels outside the boundaries were labeled as “No-Copper mine.” The manually delineated boundaries were overlaid with the classification results to count the number of pixels in each category, which were then used to construct the confusion matrix.

The resulting confusion matrix (Table 2) indicated an overall accuracy of 91.08%, reflecting the proportion of correctly classified pixels across all categories and demonstrating the model’s strong overall performance. The user’s accuracy for copper mine areas was 92.64%, indicating the likelihood that areas classified as copper mines were correctly labeled. The producer’s accuracy was 91.76%, representing the probability that actual copper mine areas were correctly identified by the model. These metrics together reflect high classification precision and recall. The Kappa coefficient of 0.8178 further confirms the statistical significance of the model’s performance by accounting for the possibility of agreement occurring by chance. A Kappa value above 0.8 is generally interpreted as a strong level of agreement, reinforcing the model’s reliability in accurately mapping copper mining areas on a global scale.

**Table 2:** Confusion Matrix and Accuracy Statistics of the Global Copper Mine Remote Sensing Identification Model.

	Copper mine	No-Copper mine	Use’s accuracy
Copper mine	2685954	213471	92.64%
No-Copper mine	241157	1955374	89.02%
Producer’s accuracy	91.76%	90.16%	

Overall accuracy = 91.08%, Kappa = 0.8178.

### Comparison with existing datasets

To contextualize the classification accuracy and spatial consistency of our model, we compared our results with existing global copper mine datasets that were developed using manual visual interpretation methods. These datasets typically rely on multi-source satellite base maps and expert delineation to define mining boundaries. While such approaches are valuable for broad-scale assessments, they often exhibit temporal inconsistency and limited spatial precision due to variations in image acquisition dates, subjective interpretation criteria, and the absence of standardized classification protocols. In contrast, our machine learning-based approach offers improved consistency and objectivity in delineating mine-specific land-use areas across global sites.

**Fig. 8:**
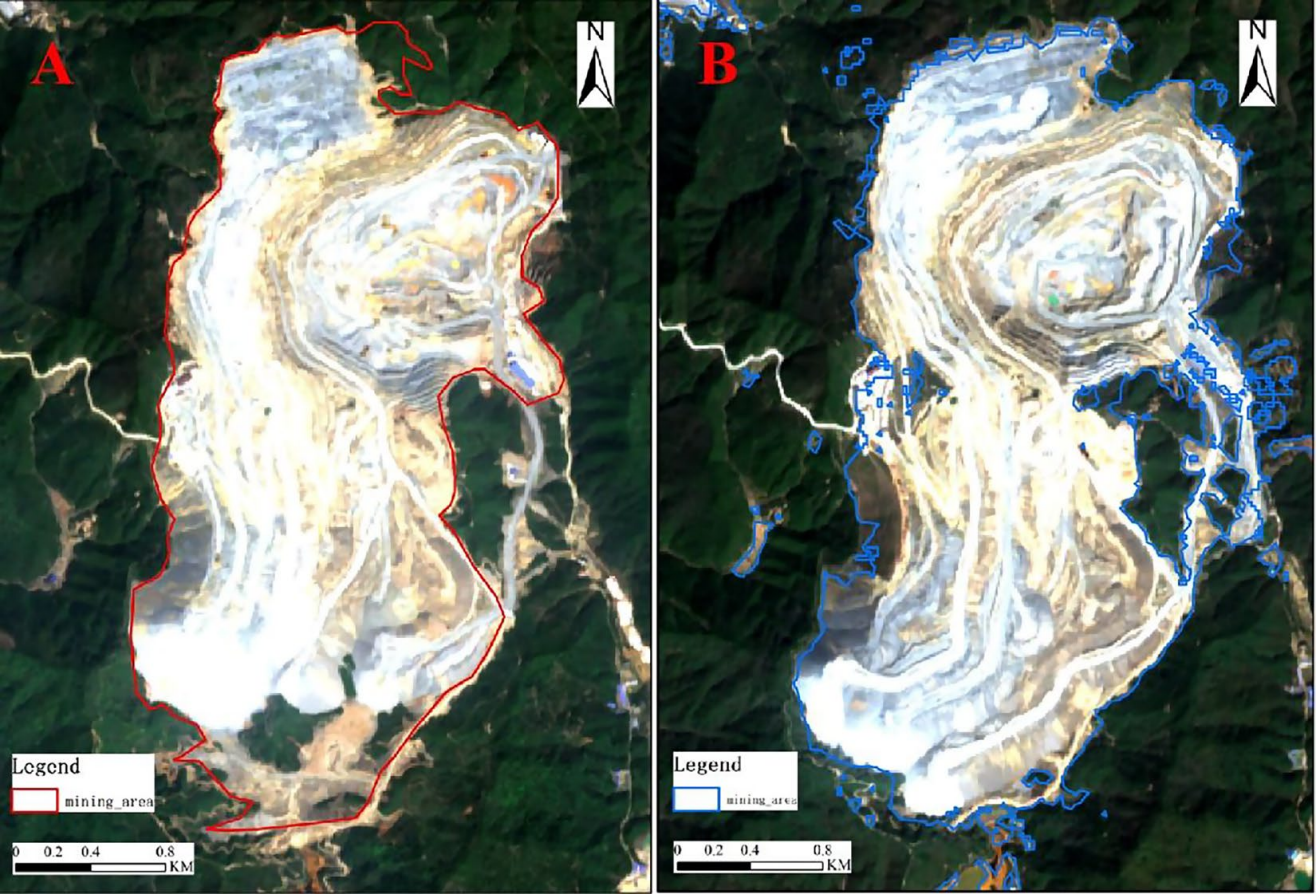
The time variation between remote sensing results and visual delineation results (**A**) for visual delineation data, Sentinel-2 image date 9 October 2017; (**B**) remote sensing extracted data, Sentinel-2 image date 12 May 2022; approx. location 28° 59′ 52.64″ N, 177° 45′ 17.87″ E.

In previous studies, the images of mining areas were plotted using base maps from software like Google Earth. These base maps are stitched together from rocker data sources of different periods and origins, which cannot guarantee the temporal consistency of all remote sensing images. Therefore, previous da’tabases cannot reflect all copper mine boundaries in the same development period. Figure 3 shows the Dexing Copper Mine, where Fig. 8(A) represents Tang & Werner’s database^25^, depicting the period of the mining area boundaries as 2017; Fig. 8(B) is our database, where all mining area boundaries are in 2022, ensuring the temporal consistency of all mining area boundaries in our data.

**Fig. 9:**
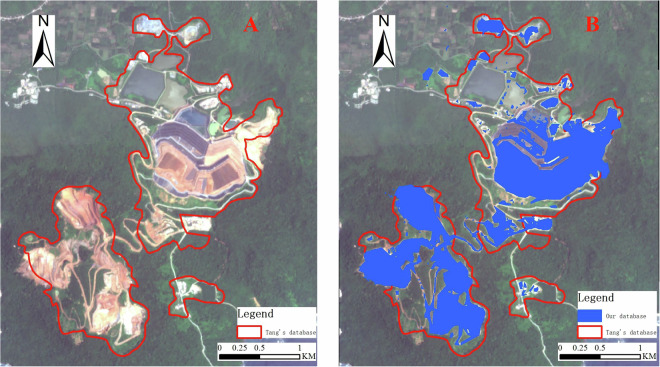
Comparison between remote sensing results and visual delineation results. (**A**) for visual delineation data (**B**) remote sensing extracted data.(Sentinel-2 image date 9 February 2022; approx. location 8° 36′ 18.59″ S, 114° 3′ 18.59″ E).

In previous databases, the delineation of mining area boundaries heavily relied on visual interpretation by professionals, a significant amount of subjective judgment from professionals was involved in the determination of mining area boundaries. As a result, besides the land use specific to mining areas, other features were also included within the mining area boundaries, as shown in Fig. 9(A). We can clearly see that a large amount of vegetation was delineated within the mining area land use. To accurately extract the land use for copper mining, it is essential to more precisely exclude this vegetation. Our database takes into account the aforementioned situation, as shown in Fig. 9(B), and more accurately delineates the land use areas for copper mining operations. Similarly, our model is not without limitations. While it accurately differentiates between mining areas and the surrounding vegetation, it demonstrates certain constraints in the separation of mine ponds.

These comparisons reinforce the strengths of our machine learning-based approach in producing mining land-use boundaries with higher spatial and temporal precision. This ensures greater utility for downstream environmental assessments and mining impact analyses.

### Sources of classification errors

Although the overall accuracy of our remote sensing classification reached 91.08%, several factors contribute to the classification errors observed:

- Spectral similarity: Some copper mine features (e.g., waste rock dumps) exhibit spectral characteristics similar to surrounding bare soils or rock outcrops, especially in arid and semi-arid regions, causing occasional misclassification.
- Mixed pixels: Due to the 10 m spatial resolution of Sentinel-2 imagery, mixed pixels at the boundary between different land-use types are inevitable, leading to uncertainties in pixel classification at feature edges.
- Cloud and shadow residuals: Despite the median composite method used to minimize cloud interference, some residual cloud shadows or atmospheric disturbances still occasionally affect the accuracy of classification.
- Vegetation disturbances: Regions with dense vegetation around mines, particularly in tropical areas, increase classification complexity, as disturbances caused by mining activities (such as roads or small clearings) can be misclassified as mining areas.

Future work could further improve accuracy through the integration of higher resolution imagery or multi-temporal classification approaches to mitigate these sources of error.

## Advantages and Applications of the Database

### Comparative advantages over existing datasets

Our database advances prior mining data resources through three key innovations:

1. Multidimensional Data Integration: Unifies high-resolution spatial footprints (10 m Sentinel-2 derived land use classifications) with operational metadata (historical production, intensity metrics), enabling cross-scale analyses that single-source datasets cannot support. This integration resolves critical gaps in correlating environmental footprints with mining productivity.
2. Machine Learning-Optimized Feature Specificity: Overcomes resolution limitations of global land cover products (e.g., MODIS 500 m) by employing a Random Forest model to map mine-specific land use categories (open pits, tailings facilities, waste dumps) at 10 m resolution. Validation via spatially stratified sampling confirmed 91.08% overall accuracy.
3. Temporally Consistent Baseline: Provides globally synchronized 2022 snapshots of mining boundaries (Fig. 3), eliminating temporal mosaicking artifacts inherent in manual databases. This consistency supports reliable time-series analyses for monitoring land degradation or rehabilitation efforts.

### Broader implications

This database, provided in vector format, offers detailed delineations of copper mining areas, historical production data, and mining intensity. It can directly support various research fields and practical applications, such as:

1. Environmental Impact Assessment: Researchers can use the detailed site-specific information to assess the ecological footprints of copper mining, particularly examining the impacts on biodiversity hotspots, carbon storage areas, and sensitive ecosystems.
2. Sustainability and Supply Chain Studies: The integrated production intensity data facilitate comparative analyses of resource efficiency and sustainability performance across global mining operations.
3. Policy and Management Applications: Policymakers and mine operators can leverage the database for effective land use planning, ecological restoration projects, regulatory compliance monitoring, and informed decision-making for licensing new mining projects or managing existing sites.

## Usage Notes

We provide a database in the form of vector data. This database includes not only the land use area and boundaries of copper mines obtained through remote sensing methods but also the cumulative production data and mining intensity per 100 square meters that we have acquired and estimated. Additionally, the database contains other descriptive information about the copper mines, such as geographic information, commodity details, and owner information,etc.

The field information in the the database is as follows:

- Name: Copper mine name
- Latitude: The latitudinal coordinates of the spatial location of the copper mine
- Longitude: Longitude coordinates of the spatial location of the copper mine
- P_C: The primary commodity of copper mines
- List_of_C: List of commodities from copper mines
- A_S: The activity status of the copper mine
- State/Prov: The state or province where the copper mine is located
- Country: The country in which the copper mine is located
- Land_use: Land use of the copper mine, measured in square meters
- Cum_Prod: Cumulative production of copper mines, measured in tons
- MI: The mining intensity of copper mines per unit area, measured in 100 m^2^/t.

## Data Availability

This paper presents the remote sensing classification model code for extracting land use in mining areas. The code is designed to be executed on the Google Earth Engine (GEE) platform and is written in JavaScript. All code and dataset are available in the figshare repository: “Machine learning-enhanced monitoring of global copper mining areas” (10.6084/m9.figshare.28680863)^49^.
