# Correction: Machine learning-enhanced monitoring of global copper mining areas

**DOI:** 10.1038/s41597-025-05594-5

**Published:** 2025-07-24

**Authors:** Houxuan Li, Peng Wang, Tim T. Werner, Bin Chen, Wei-Qiang Chen

**Affiliations:** 1 Institute of Urban Environment, Chinese Academy of Sciences, 1799 Jimei Road, Xiamen, 361021 China; 2 School of Geography, University of Melbourne, 221 Bouverie Street, Carlton, VIC 3010 Australia; 3 Department of Environment Science and Technology, Fudan University, 220 Handan Rd., Shanghai, 200433 China

**Keywords:** Environmental monitoring, Environmental impact

Correction to: *Scientific Data* 10.1038/s41597-025-05296-y, published online 01 July 2025

In this article, figure 5 omitted the colour rendering for some regions in China due to a technical oversight during figure preparation. The original article has been corrected.

Original figure 5

Corrected figure 5

